# Two case reports of distal upper limb weakness following influenza-like illness: an emerging pattern of para-infectious myositis in adults

**DOI:** 10.1186/s12883-020-01821-1

**Published:** 2020-06-10

**Authors:** Jakub Scaber, Adam J Molyneux, Camilla Buckley, Alastair J S Webb

**Affiliations:** 1 Nuffield Department of Clinical Neurosciences, University of Oxford, West Wing Level 6, John Radcliffe Hospital, Headley Way, Oxford, OX3 9DU UK; 2 Department of Clinical Neurophysiology, Oxford University Hospitals, Foundation Trust, Oxford, UK

**Keywords:** Para-infectious, Muscle disease, Myositis, Case report

## Abstract

**Background:**

Myositis is a recognised complication of numerous systemic viral infections including influenza. In adults the typical pattern is characterised by myalgia and marked proximal muscle weakness in upper and lower limbs and resolves slowly over weeks rather than days.

**Case presentation:**

Here, we describe two male patients with myositis with an unusual distribution of weakness in the distal upper limbs, which both followed a flu-like illness and resolved spontaneously. Both patients had moderate elevations in creatine kinase, extensive negative serological investigations, normal nerve conduction studies and myopathic changes on electromyography.

**Conclusions:**

In the para-infectious context, myositis is an important differential of acute distal upper limb weakness. This unusual pattern of acute muscle weakness should be recognised to avoid unnecessary in treatments. Similar cases in the recent literature in male patients between the ages of 25 to 55 are reviewed and suggest an emerging pattern of para-infectious myositis.

## Background

Myositis is a recognised complication of numerous systemic viral infections including influenza [1]. In adults the typical pattern characterised with myalgia and marked proximal muscle weakness in upper and lower limbs and resolves slowly over weeks rather than days [2].

Here, we describe two patients with myositis with an unusual distribution of weakness in the distal upper limbs, which both followed a flu-like illness and resolved spontaneously. We review similar cases in the recent literature and postulate a new form of para-infectious myositis.

## Case presentation

### Case 1

A 38-year-old medical professional with no relevant past medical history presented with a four-day history of fevers and rigors. On day three, he complained of neck pain and loose stools after taking amoxicillin. On day four, he awoke with difficulty using both hands and difficulty ascending stairs, as well as pain in his forearms and thighs. By the evening, the symptoms had progressed, and he was unable to hold cutlery, and complained of numbness in his fingertips.

**Fig. 1:**
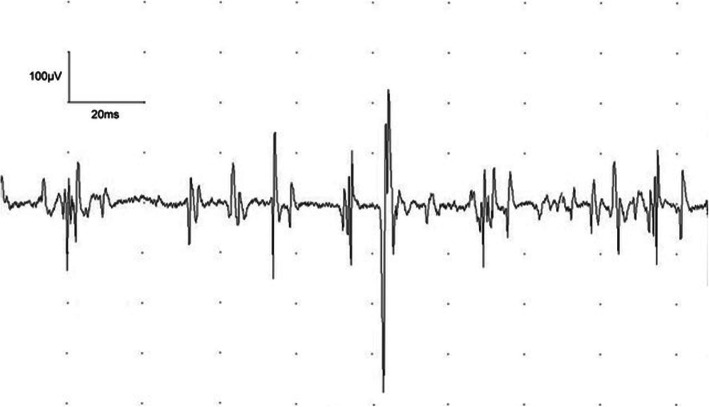
Representative EMG showing myopathic changes evidenced by multiple polyphasic motor unit action potentials (MUAPs), which are of small amplitude (< 0.5 mV), and some of which are of short duration (5–10 ms)

Examination showed weakness of finger flexion (grade 4) and extension bilaterally (grade 4), with relative preservation of abduction (grade 4+). There was also mild weakness of hip flexion bilaterally (grade 4+). All remaining upper and lower limb muscles were normal (grade 5). Reflexes were present and pinprick sensation was reduced in the distribution of the C6 dermatome bilaterally. Magnetic resonance imaging (MRI) of the whole spine and brachial plexus was normal. Creatine kinase (CK) was 2352 IU/L on admission, but blood tests were otherwise normal including a CRP of 2.8 mg/L and a negative blood culture, and lumbar puncture was acellular with a protein of 237 mg/L and normal glucose. Acutely, random serum glucose was 13 mmol/L, but subsequently normalised. Nerve conduction studies were normal but electromyography (EMG) a day after admission showed mild abnormalities of the forearm muscles suggestive of myopathy (Table 1 and Fig. 1). The patient improved with return of normal arm power and walking after 48 h. A muscle biopsy was not felt to be required given the improvement. The CK initially rose to 2703 but this improved to 765 a week later. Extensive serologic testing during the admission as well as influenza swabs were negative (Table 2).

**Table 1:** Electrophysiologic findings

	Patient 1				Patient 2			
**Sensory Studies**	**Lat**	**Amp**	**Velocity**		**Lat**	**Amp**	**Velocity**	
	**ms**	**μV**	**m/s**		**ms**	μV	**m/s**	
R Median Digit 2	2.35	17.8	66		2.75	7.8	52.7	
R Median Digit 3					3.05	7.5	47.5	
R Ulnar Digit 5	2.05	7.5	63.4		2.05	5.5	56.1	
R Median Palm (Mix)					1.65	23.6	48.5	
R Ulnar Palm (Mix)					1.35	5.2	59.3	
L Median Digit 2					2.9	6.8	51.7	
L Median Digit 3					2.7	8.6	55.6	
L Ulnar Digit 5					2.25	6.1	51.1	
R Sural Calf	2.1	12	47.6		2.7	12.4	40.7	
L Sural Calf					2.85	11.5	45.6	
R Superficial Peroneal					2.75	7.8	41.8	
L Superficial Peroneal					3.05	5.7	42.6	
**Motor Studies**	**Lat**	**Amp**	**Velocity**	**F Lat**	**Lat**	**Amp**	**Velocity**	**F Lat**
	**ms**	**mV**	**m/s**	**ms**	**ms**	**mV**	**m/s**	**ms**
R Median Wrist	2.8	13.1			3.25	10.6		
R Median Elbow	7	13.2	59.5	26.1	7.85	9.9	60.9	28.6
L Median Wrist	2.8	10.9			3.15	11.2		
L Median Elbow	6.65	11.1	65	26.05	7.95	11	56.3	28.6
R Ulnar Wrist	2.4	10.7			2.45	13.9		
R Ulnar Below Elbow	5.85	10	75.4	26.05	6.8	14.5	57.5	
R Ulnar Above Elbow					8.35	12.8	51.6	31.6
L Ulnar Wrist	2.25	6.7			2.55	8.2		
L Ulnar Below Elbow	5.95	6.2	69.2	26.55	6.85	8.2	58.1	30.5
L Ulnar Above Elbow					8.5	7.9	54.5	
R Com Peroneal Ankle	3.2	7.1			4.25	9.2		
R Com Peroneal Fib Head	9.45	6.3	56	41.7	12.45	8.7	42.7	54.45
R Com Peroneal Knee	10.65	6.5	66.7		14.2	8.3	45.7	54
L Com Peroneal Ankle					4.25	8.1		
R Post Tibial Ankle	4.75	18.8			4	15.7		
R Post Tibial Knee	11.8	15.8	58.2	44.9	14.05	12.6		52.75
L Post Tibial Ankle					3.6	10.5		
L Post Tibial Knee					13.95	8.7	45.4	55.6
**EMG**	**MUAP**			**Recruit Pattern**	**MUAP**			**Recruit Pattern**
	**Amp**	**Dur**	**Polyph**		**Amp**	**Dur**	**Polyph**	
R Triceps					N	N	N	N
R Extensor Dig Comn	N	N/1-	N/1+	N/Fast	1-	N	1+	Fast
R Flexor Dig Sup					N	N	1+	N
R First Dorsal Interos	N	N	N	N	1-	N	N	N
L Flexor Dig Sup	N	N/1-	N/1+	N/Fast				
L Flexor Dig Prof IV	N	N/1-	N/1+	N/Fast				
R Vastus Medialis					1-	N	N	N
R Tibialis Anterior					1-	N	N	Fast

Table legend: Electrophysiologic findings for patients 1 and 2. There was no spontaneous activity on EMG in either of the two patients. *EMG* electromyography, *Lat* latency, *Amp* amplitude, *F Lat* minimum F wave latency, *Mix* mixed nerve, *N* normal, *L* left, *R* right, *Dur* duration, *Polyph* polyphasia, *MUAP* motor unit action potential, *Com* common, *Comn* communis, *Fib* fibular, *Post* posterior, *Dig* digitorum, *Sup* superficialis, *Prof* profundus, *Interos* interosseus, *Recruit* recruitment

**Table 2:** Laboratory investigations

	Patient 1	Patient2
Influenza A/B & RSV PCR	–	–
CMV IgG	–	+
CMV IgM	–	–
EBV IgG	+	+
EBV IgM	–	–
VZV IgG		+
Hepatitis B serology	Immunised	–
Hepatitis C serology	–	–
Hepatitis E IgG	–	+
Hepatitis E IgM	–	–
HIV serology	–	–
Parvovirus IgG	+	
Parvovirus IgM	–	
Borrelia serology	–	–
Borrelia CSF line blot		–
Mycoplasma serology	–	–
Syphilis serology		–
Antistreptolysin O titre		–
Myositis panel	–	
GM1/GQ1b	–	–
Antinuclear Antibodies	–	–

Table legend: Pertinent laboratory results for patients 1 and 2. *CMV* cytomegalovirus, *CSF* cerebrospinal fluid, *EBV* Eppstein-Barr virus, *HIV* human immunodeficiency virus, *RSV* respiratory syncytial virus, *VZV* varicella zoster virus

### Case 2

A 54-year-old man presented with a four-day history of fevers above 40 °C, rigors and sweats. On day three he developed leg weakness, struggling to climb stairs and getting out of the bath. There was associated thigh tenderness. Later that day, he also developed hand weakness, being unable to open bottles. On day four he required help to dress himself. There were no autonomic symptoms or sphincter disturbance and no sensory symptoms other than mild paraesthesia in the fingertips of both hands. He complained of mild neck pain.

Examination revealed distal more than proximal upper and lower limb weakness, particularly of finger flexion (grade 4 left, grade 4+ right) and extension (grade 4 bilaterally). There was also some weakness of hip extension (grade 4). Otherwise there was mild weakness in all muscle groups tested in the upper and lower limbs bilaterally (grade 4+), except for wrist extension and hip flexion, which were both normal (grade 5). Reflexes were present and sensation was normal. Computed tomography (CT) of the head was normal. Admission blood tests demonstrated a CK of 987 U/L and CRP of 6.9 mg/L and were otherwise normal. Cerebrospinal fluid (CSF) was acellular, with a protein of 484 mg/L and normal glucose. His condition improved spontaneously over the next 3 days and his CK returned to 180 U/L, within the normal range. After another 5 days CK fell further to 80 U/L. Muscle biopsy was therefore deemed not to be required. EMG a week after presentation showed subtle myopathic changes, most convincing from the tibialis anterior and extensor digitorum communis, nerve conduction studies were normal. Influenza swab and extensive serologic testing during the admission was negative in this case also (Table 2).

## Discussion and conclusions

The two cases presented here demonstrate clinical and laboratory studies consistent with a myopathic process, but with an atypical muscle pattern and predominant upper limb distal weakness. In both cases, no specific pathogen could be isolated. These two presentations occurred months apart and both patients recovered to full function within days to weeks. The main differential diagnosis considered initially in both cases was Guillain-Barré syndrome (GBS), but the clinical and laboratory characteristics favoured a diagnosis of myositis for both patients. Given the mild sensory changes in the context of normal nerve conduction studies, we cannot completely exclude subclinical peripheral nerve involvement, and mild irritation of the lateral cutaneous nerve of the forearm by forearm muscle swelling or proximal radicular demyelination could have theoretically co-occurred but are not proven.

Benign acute myositis is well-recognised as a childhood illness, where it predominantly involves the calf muscles and is associated with influenza B virus. In adults, viral and post-infectious myositis is rarer but has been described in the context of influenza, mononucleosis, cytomegalovirus infection, echovirus 9 and viral hepatitis [1]. Cases share a characteristic pattern of prodromal myalgia followed by proximal muscle weakness, muscle tenderness, myoglobinuria and a slow recovery that is often incomplete by the time of discharge.

A novel pattern of distal pattern upper limb weakness following an influenza-like prodrome was first described during the H1N1 2009 influenza epidemic in Utah, with one of the cases also being a medical professional [3]. A similar case was reported in December 2017 in the UK [4]. There multiple are striking similarities between the four previously reported cases and the two cases presented here: All are men between the age of 25 and 54, with muscle tenderness CK elevations ranging from 500 to 3500. While all six patients had notable weakness particularly of finger flexion and extension, four patients also had demonstrable proximal lower limb weakness, which was usually proximal. A febrile illness was reported to have preceded weakness by 2–5 days in all cases except one, who reported flu-like symptoms starting a few weeks earlier. The weakness recovered to normal or near normal within 1 week from the start of the symptoms in the majority of patients.

Analogous to previous reports, we could not confirm an associated infectious agent in our patients on extensive serologic testing. The first patient presented towards the tail end of the seasonal influenza epidemic, with the prevalent strains being B and A(H3), while the second patient presented in the summer when influenza was not circulating in significant quantities. Although negative for respiratory viral PCR, all cases thus far had occurred during H1N1 influenza outbreaks, which led to the hypothesis that the pattern of distal upper limb weakness may be specific to this strain of influenza. This is unlikely given the timing of our cases. We hypothesize that a viral pathogen is the most likely explanation for the influenza-like febrile illness with a normal or only very mildly raised CRP in both patients. Given the very short time of 3 days between the onset of the flu-like illness and the onset of neurological symptoms, a direct effect of the virus on muscle cells causing necrosis [5] or an effect of the immune response to the virus is the most likely explanation [1]. The absence of a latency period means that a triggered secondary immune response, as is seen in post-infectious diseases such as GBS, is unlikely [6].

The cases reported here provide further evidence for an emerging pattern of para-infectious myositis, unusually involving the distal upper limbs. Myositis should therefore be considered by clinicians seeing patients with wrist and hand weakness following influenza-like illness. Confident recognition of this muscle pattern and distinction from commoner causes of acute distal upper limb weakness, such as Guillain-Barre syndrome and inflammatory myopathies, is paramount to avoid unnecessary investigations or treatments including muscle biopsies, steroids or intravenous immunoglobulin [7]. Treatment for para-infectious myositis is symptomatic and renal function should be monitored alongside CK and managed appropriately.

## Data Availability

Not applicable.

## Abbreviations

ANA: Antinuclear antibodies
CMV: Cytomegalovirus
CRP: C reactive protein
CSF: Cerebrospinal fluid
EBV: Eppstein-Barr virus
GBS: Guillain-Barré syndrome
HIV: Human immunodeficiency virus
PCR: Polymerase chain reaction
RSV: Respiratory syncytial virus
VZV: Varicella zoster virus
